# Anxiety–Depression of Dialysis Patients and Their Caregivers

**DOI:** 10.3390/medicina55050168

**Published:** 2019-05-20

**Authors:** Georgia Gerogianni, Maria Polikandrioti, Fotoula Babatsikou, Sofia Zyga, Victoria Alikari, George Vasilopoulos, Stavroula Gerogianni, Eirini Grapsa

**Affiliations:** 1 Department of Nursing, University of West Attica, 11528 Athens, Greece; mpolik2006@yahoo.com (M.P.); fibaba@uniwa.gr (F.B.); vicalikari@gmail.com (V.A.); gvasilop@teiath.gr (G.V.); 2 Department of Nursing, University of Peloponnese, 23100 Sparta, Greece; zygas@uop.gr; 3 Department of Nursing, ‘Alexandra’ Hospital, 15772 Athens, Greece; g.roula80@gmail.com; 4 Department of Nephrology, ‘Aretaieio’ Hospital, National and Kapodistrian University of Athens, 157 72 Athens, Greece; egrapsa@aretaieio.uoa.gr

**Keywords:** anxiety, depression, patients, caregivers, end stage renal disease, hemodialysis, kidney failure

## Abstract

*Background and Objectives:* Anxiety–depression of patients undergoing hemodialysis has a strong relation with the levels of anxiety–depression of their caregivers. The aim of this study was to evaluate anxiety–depression of dialysis patients and their caregivers. *Materials and Methods:* In this cross-sectional study, 414 pairs of patients and caregivers from 24 hemodialysis centers of Greece completed the Hospital Anxiety and Depression Scale (HADS). The statistical analysis of the data was performed through the Statistical Program SPSS version 20.0. The statistical significance level was set up at 5%. *Results:* The mean age of patients was 64 (54.06–72.41) years old and the mean duration of hemodialysis was 36 (16–72) months. The mean age of caregivers was 54 (44–66) years old. Of the total sample, 17.1% (*n* = 71) of patients had high levels of anxiety and 12.3% (*n* = 51) had high levels of depression. Additionally, 27.8% (*n* = 115) of caregivers had high levels of anxiety and 11.4% (*n* = 47) had high levels of depression. Caregivers had higher levels of anxiety when their patients had high levels of anxiety as well (42.3%). Additionally, they had higher levels of depression when their patients had high levels of depression as well (17.6%). *Conclusions*: The results of this study showed a significant association between the levels of anxiety and depression among patients and caregivers. There is a necessity for individualized assessment of dialysis patients and their caregivers and the implementation of specific interventions for reducing the levels of anxiety and depression among them.

## 1. Introduction

Anxiety and depression are common psychiatric disorders among patients undergoing hemodialysis (HD) [1,2] and their caregivers [3,4]. Several factors seem to trigger anxiety and depression in hemodialysis patients such as co-morbidities, frequent hospitalizations [5], chronic pain, sleep disturbances [6], chronic inflammation, increased fatigue, decreased sexual functioning [7], uremia [8], failure of family support restrictions in daily life, non-compliance to therapeutic regimen including restrictions in diet and fluids, and dependency upon treatment and health professionals [9].

Given that a disease not only affects a member of the family but also dynamics within the family, the prevalence of anxiety and depression among caregivers is easily understandable. Dialysis therapy imposes several restrictions in caregivers’ life such as decreased physical function, fatigue, social isolation, difficulties in relationships [10,11], and feelings of disappointment [12]. It is worthy to note that advancing age of caregivers significantly complicates all the above limitations [4]. More in detail, in terms of gender, women caregivers seem to be more vulnerable to anxiety and depression, mainly attributed to their role in family and children care [13].

Interestingly, health care professionals have the tendency to focus on the biological dimension of the disease or other technical issues related to hemodialysis machine and usually underestimate symptoms from mental sphere. Encouraging patients to express their feelings and addressing their psychological needs may be an essential measure to confront with this debilitating disease [9]. An underestimated or untreated anxiety and depression may lead to diminished quality of life among both patients and caregivers [14].

To the best of our knowledge, research exploring effects of anxiety and depression between patients and caregivers is limited. Thus, the aim of this cross-sectional study was to explore the effect of anxiety and depression of dialysis patients on anxiety and depression of their caregivers.

## 2. Materials and Methods

### 2.1. Study Participants

From June 2016 to February 2017, a sample of 805 pairs of patients and their caregivers were recruited from 24 dialysis centers in Athens and Thessaloniki, Greece. Athens and Thessaloniki are the most populated cities of Greece. Inclusion criteria for patients were age over 18 years old and less than 85 years old, on HD three times a week for at least three months, and the ability to speak, read, and write in Greek. Exclusion criteria were previous treatment for depression, inadequate language skills, age over 85 years and less than 18 years old, cognitive dysfunction, and drug or alcohol abuse. The cognitive dysfunction was assessed by the clinical judgment made by the dialysis staff. Inclusion criteria for caregivers were being spouses, parents, daughters, or sons of the patients while exclusion criteria were individuals who were taking care of patients with payment. Finally, the study sample included 414 (response rate 51.4%) pairs of patients and caregivers after receiving written informed consent from each participant. Participants were approached during their routine treatment.

Participants were provided with a verbal description of the study purpose and procedure, while they were asked to complete all the questionnaires at that time. Before collecting data, we obtained approval from the Ethics Committee of National and Kapodistrian University of Athens, Department of Medicine, “Aretaieio” Hospital.

The data included gender, age, occupation, nationality, residence, education, marital status, number of children, financial situation, duration of HD, co-morbid diseases, escort to the dialysis unit, and transplant candidate.

### 2.2. Data Collection

#### Hospital Anxiety and Depression Scale (HADS)

Anxiety and depression were evaluated by the hospital anxiety and depression scale (HADS), which contained 14 questions and was recommended by Zigmond and Snaith [15]. Specifically, seven of those questions (2, 4, 6, 8, 10, 12, and 14) estimate the degree of depression and the rest of them (1, 3, 5, 7, 9, 11, and 13) estimate the degree of anxiety. A Likert scale from 0 to 3 is used to answer each question. Scores are separately added for anxiety and depression. The whole score ranges from 0 to 21.

Additionally, the literature indicated the following rating as well: 0–7 indicates lack of anxiety or depression, 8–10 indicates average levels of anxiety or depression, and score more than 11 shows increased levels of anxiety or depression. HADS had high reliability and validity in Greece, especially in cancer patients [16,17].

### 2.3. Data Analysis

Categorical data are presented in absolute and relative (%) frequencies, while continuous data are presented with median and interquartile range (IQR). Normality was tested with Kolmogorov–Smirnov criterion and graphically with Q–Q plots and did not hold. Non-parametric tests were used to evaluate the association between variables. Multinomial logistic was performed to estimate the effect of patients’ anxiety and depression on that of the caregivers after controlling for potential confounders. Results are presented with odds ratio (OR) and 95% confidence interval (CI). The observed level of significance was set up to 5%. All statistical analyses were performed with SPSS version 20 (SPSS Inc., Chicago, IL, USA).

## 3. Results

### 3.1. Sample Description

Of the total 414 pairs of patients and caregivers, 63.3% (*n* = 262) of patients were men while 76.3% (*n* = 316) of caregivers were women. The median age of patients was 63.54 years old and the median duration of HD was 36 months. The median age of the caregivers was 54 years old. The majority of patients 70.5% (*n* = 292) and 72.7% (*n* = 301) of caregivers were married. Of the total sample, 45.2% (*n* = 187) of patients and 47.1% (*n* = 195) of caregivers had secondary education. In total, 75.8% (*n* = 314) of patients and 30.4 % (*n* = 126) of caregivers were pensioners. Additionally, 56% (*n* = 232) of patients and 59.4% (*n* = 246) of caregivers had moderate financial condition. Totally, 66.8% (*n* = 276) of patients and 33.1% (*n* = 137) of caregivers had another disease. The majority of caregivers were husbands/wives—56.3% (*n* = 233) and children—25.4% (*n* = 105) (Table 1).

### 3.2. Description of the HADS Categories

Of the total sample, 17.1% (*n* = 71) of patients had high levels of anxiety and 12.3% (*n* = 51) had high levels of depression. Additionally, 27.8% (*n* = 115) of caregivers had high levels of anxiety and 11.4% (*n* = 47) had high levels of depression. Regarding the comparison between patients and caregivers, caregivers had statistically significant higher levels of anxiety in the HADS than patients (27.8% versus 17.1% *p* < 0.001) (Table 2).

### 3.3. Association of Patients’ and Caregivers’ Anxiety

There is a statistically significant association between patients’ anxiety levels and those of caregivers (*p* = 0.008). Caregivers had higher levels of anxiety when their patients had high levels of anxiety as well (42.3%). Additionally, they had higher levels of depression when their patients had high levels of depression as well (17.6%) (Table 3).

In HADS, multinomial logistic regression revealed that caregivers who had patients with high levels of anxiety had 2.54 times higher probability, than those who had patients with low levels of anxiety, to have high levels of anxiety compared to low levels, after adjustment for potential confounders (OR = 2.54, 95% CI: 1.33–4.83, *p* = 0.005). Similarly, caregivers who had patients with moderate depression levels had a 2.44-fold higher probability, than those who had patients with low depression, to have moderate levels of depression compared to low levels, after adjusting for potential confounders (OR = 2.44, 95% CI: 1.23–4.82, *p* = 0.010). In addition, caregivers who had patients with high levels of depression have a 4.27- and 3.46-fold greater probability, than those who had patients with low depression, to have moderate and high levels of depression, respectively, compared to low levels, after adjustment for potential confounding factors (OR = 4.27, 95% CI: 2.02–9.04, *p* = 0.001 and OR = 3.46, 95% CI: 1.28–9.31, *p* = 0.014, respectively) (Table 4).

## 4. Discussion

According to the results of the present study, 17.1% and 12.3% of patients experienced high levels of anxiety and depression, respectively, while 27.8% and 11.4% of caregivers experienced high levels of anxiety and depression, respectively. More importantly, caregivers had high levels of anxiety and depression when their patients had high levels of anxiety and depression as well.

The results of the present study are in line with other similar studies that showed depression rates among dialysis patients between 19% and 60% [18,19]. More specifically, in 395 Greek patients (222 men and 173 women) undergoing hemodialysis, 47.8% experienced high levels of anxiety and 38.2% high levels of depression. [9]. Depression levels among patients on hemodialysis are similar to those of cancer patients since chronic kidney disease, although is not itself a fatal disease, causes great upsets in the daily routine and the quality of life among patients and their families [20]. On the other end of the spectrum, caregivers are 4 times more likely to have depressive symptoms and 3 times more likely to receive therapy for anxiety disturbances compared to individuals not providing care [21].

The results of the current study regarding caregivers’ anxiety and depression are in line with other relevant studies, demonstrating that caring for patients undergoing hemodialysis exerts a great negative effect on their emotional state. More in detail, caregivers experience anxiety, depression, physical and mental fatigue, deterioration in family relationships, and social isolation [21,22,23,24]. According to Zyada et al. [22], changes in caregivers’ plans about future or expectations may partially explain this emotional burden. Nipp et al. [21] supported that levels of anxiety and depression in caregivers of patients undergoing hemodialysis may be similar to those involved in care of cancer using HADS.

The current findings concerning the association between anxiety and depression among patients and their caregivers seems to be consistent with literature. Several explanations may account for this observed association. For instance, an untreated anxiety may lead to depression [25], and may have a negative effect on interpersonal relationships, thus leading to failure in adapting to demands of this chronic treatment [26,27]. An alternative conclusion is that the type of personality may provoke anxiety and depression among patients and caregivers [28].

Additionally, a prior study by Khaira et al. [29] showed high levels of depression among patients were associated with high levels of depression among spouses. Therefore, exploring this depressive dyad is a new significant area related to treatment of hemodialysis [29]. In long term, poor caregivers’ psychological status is associated with increased patients’ mortality, for the reason that depressed caregivers frequently neglect the needs of their loved persons, thus leading in a diminished quality of life [20,30]. Saeed et al. [31] exploring 180 patients and 180 caregivers showed that 75% of patients and 33.4% of caregivers experienced moderate to severe depression with marital status and low economic state to be associated with high levels of depression. Given that the number of patients undergoing hemodialysis is expanding at an alarming rate, it is obvious that the burden of caregivers is expected to increase.

The present results put an emphasis on evaluating caregivers’ psychological well-being. A better understanding of the association between patients’ and caregiver’s anxiety and depression may help in planning for future and effective interventions among this vulnerable population. Though considerable advances were made during recent years in understanding psychiatric disorders among hemodialysis patients, caregivers may need closer examination or a specific and constant diagnostic evaluation. Support provided by family has been broadly linked to improved health outcomes in chronic illness, independent of geographic settings and ethnic backgrounds. Therefore, healthy caregivers are a valuable resource for patients. Educational interventions that target at caregivers’ needs represent a new area of interest and may markedly decrease the economic, medical, individual, and social burden of hemodialysis [32].

### Limitations of the Study

Several limitations of our study must be acknowledged. First, convenience sampling is one of the limitations as this method is not representative of all population undergoing hemodialysis in Greece, thus limiting the generalizability of results. Furthermore, there was no other measurement in time that would display changes in anxiety/depression over time. Additionally, a control group of patients and caregivers was not included. Finally, anxiety and depression were assessed using self-report, and no information on an established clinical diagnosis was collected.

The strengths of the study include the use of a wide spread analysis “HADS” that may permit comparisons among hemodialysis populations and caregivers across the world.

## 5. Conclusions

The present study showed that caregivers had high levels of anxiety and depression when their patients had high levels of anxiety and depression as well.

A better understanding of the association between patients’ and caregivers’ anxiety and depression may help in planning and implementing future interventions or identifying effective strategies to maintain mental health among these vulnerable population.

## Figures and Tables

**Table 1:** Characteristics of participants (*n* = 414).

Patients	N (%)	Caregivers	N (%)
**Gender**		**Gender**	
Male	262 (63.3%)	Male	98 (23.7%)
Female	152 (36.7%)	Female	316 (76.3%)
**Nationality**		**Nationality**	
Greek	405 (97.8%)	Greek	394 (95.2%)
Other	9 (2.2%)	Other	20 (4.8%)
**Residence**		**Residence**	
Attica	260 (62.8%)	Attica/capital city	374 (90.4%)
Capital city	110 (26.6%)	Small town/village	40 (9.7%)
Small town	15 (3.6%)		
Village	29 (7.0%)		
**Education**		**Education**	
Primary	103 (24.9%)	Primary	67 (16.2%)
Secondary	187 (45.2%)	Secondary	195 (47.1%)
Bachelor	111 (26.8%)	Bachelor	126 (30.4%)
MSc–PhD	13 (3.1%)	MSc–PhD	26 (6.3%)
**Status**		**Status**	
Single	34 (8.2%)	Single	67 (16.2%)
Married	292 (70.5%)	Married	301 (72.7%)
Divorced	31 (7.5%)	Divorced	13 (3.1%)
Widowed	49 (11.8%)	Widowed	9 (2.2%)
Living together	8 (1.9%)	Living together	24 (5.8%)
**Children**		**Children**	
No	75 (18.1%)	No	115 (27.8%)
Yes	339 (81.9%)	Yes	299 (72.2%)
**Number of Children**		**Number of Children**	
1	79 (23.3%)	1	67 (22.4%)
2	193 (56.9%)	2	177 (59.2%)
>2	67 (19.8%)	>2	55 (18.4%)
**Living Alone**		**Living Alone**	
No	369 (89.1%)	No	387 (93.5%)
Yes	45 (10.9%)	Yes	27 (6.5%)
**Job**		**Job**	
Civil servant	13 (3.1%)	Civil servant	30 (7.2%)
Private employee	11 (2.7%)	Private employee	73 (17.6%)
Freelancer	16 (3.9%)	Freelancer	35 (8.5%)
Household	42 (10.1%)	Household	92 (22.2%)
Farmer	5 (1.2%)	Farmer	4 (1.0%)
Student	3 (0.7%)	Student	5 (1.2%)
Unemployed	10 (2.4%)	Unemployed	49 (11.8%)
Pensioner	314 (75.8%)	Pensioner	126 (30.4%)
**Financial Situation**		**Financial Situation**	
Bad	69 (16.7%)	Bad	66 (15.9%)
Moderate	232 (56.0%)	Moderate	246 (59.4%)
Good	103 (24.9%)	Good	94 (22.7%)
Very Good	9 (2.2%)	Very Good	8 (1.9%)
Perfect	1 (0.2%)	Perfect	0 (0.0%)
**Other Diseases**		**Other Diseases**	
No	137 (33.2%)	No	277 (66.9%)
Yes	276 (66.8%)	Yes	137 (33.1%)
**Escort**		**Relation with Patient**	
Alone	256 (61.8%)	Husband/Wife	233 (56.3%)
Parents	5 (1.2%)	Parent	25 (6.0%)
Children	47 (11.4%)	Children	105 (25.4%)
Consort	88 (21.3%)	Grand children	4 (1.0%)
Other	18 (4.3%)	Sibling	17 (4.1%)
**Transplant Candidate**		**Other**	30 (7.2%)
No	275 (66.4%)		
Yes	139 (33.6%)		
	**Median (IQR)**		**Median (IQR)**
**HD Duration (months)**	36 (16–72)		
**Age (years)**	63.54 (54.06–72.41)	**Age (years)**	54 (44–66)

N, sample size; IQR, interquartile range.

**Table 2:** Description of the hospital anxiety and depression scale (HADS) categories.

	Patients	Caregivers	
	N(%)	N(%)	*p*-Value
**Anxiety**			**<0** **.001**
Low levels	265 (64.0%)	199 (48.1%)	
Moderate levels	78 (18.8%)	100 (24.2%)	
High levels	71 (17.1%)	115 (27.8%)	
**Depression**			0.780
Low levels	292 (70.5%)	289 (69.8%)	
Moderate levels	71 (17.1%)	78 (18.8%)	
High levels	51 (12.3%)	47 (11.4%)	

**Table 3:** Association of patients’ and caregivers’ anxiety and depression (HADS).

**Caregivers’ Anxiety**				
	**Low Levels**	**Moderate Levels**	**High Levels**	
	**N (%)**	**N (%)**	**N (%)**	***p*-Value**
**Patients’ Anxiety**				**0.008**
Low levels	141 (53.2%)	65 (24.5%)	59 (22.3%)	
Moderate levels	33 (42.3%)	19 (24.4%)	26 (33.3%)	
High levels	25 (35.2%)	16 (22.5%)	30 (42.3%)	
**Caregivers’ Depression**				
	**Low Levels**	**Moderate Levels**	**High Levels**	
	**N (%)**	**N (%)**	**N (%)**	***p*-Value**
**Patients’ Depression**				**0** **.001**
Low levels	224 (76.7%)	42 (14.4%)	26 (8.9%)	
Moderate levels	41 (57.7%)	18 (25.4%)	12 (16.9%)	
High levels	24 (47.1%)	18 (35.3%)	9 (17.6%)	

**Table 4:** Effect of patients’ anxiety/depression on caregivers (HADS).

	**Caregivers’ Anxiety (Reference Category: Low Levels)**			
	**Moderate Levels**		**High Levels**	
	**OR (95% CI)**	***p*-Value**	**OR (95% CI)**	***p*-Value**
**Crude Regression**				
**Patients’ Anxiety**				
Low levels	Ref. Cat.		Ref. Cat.	
Moderate levels	1.25 (0.66–2.36)	0.494	1.88 (1.04–3.42)	**0.038**
High levels	1.39 (0.69–2.78)	0.353	2.87 (1.56–5.29)	**0.001**
Adjusted Regression *				
**Patients’ Anxiety**				
Low levels	Ref. Cat.		Ref. Cat.	
Moderate levels	1.06 (0.55–2.04)	0.871	1.8 (0.96–3.38)	0.067
High levels	1.18 (0.58–2.41)	0.647	2.54 (1.33–4.83)	**0.005**
	**Caregivers’ Depression (Reference Category: Low Levels)**			
	**Moderate Levels**		**High Levels**	
	**OR (95% CI)**	***p*-Value**	**OR (95% CI)**	***p*-Value**
**Crude Regression**				
**Patients’ Depression**				
Low levels	Ref. Cat.		Ref. Cat.	
Moderate levels	1.64 (0.84–3.21)	0.147	2.52 (1.37–4.63)	**0.003**
High levels	1.68 (0.8–3.54)	0.174	2.13 (1.05–4.3)	**0.036**
Adjusted Regression **				
**Patients’ Depression**				
Low levels	Ref. Cat.		Ref. Cat.	
Moderate levels	2.44 (1.23–4.82)	**0.010**	2.01 (0.87–4.66)	0.104
High levels	4.27 (2.02–9.04)	**0.001**	3.46 (1.28–9.31)	**0.014**

Ref.Cat, Reference Category; OR, Odds Ratio; * Adjusted for relation with patient, financial situation, and other disease (univariate analysis). ** Adjusted for age, relation with patient, education, job, financial situation, and other disease (univariate analysis).
